# PREST-plus identifies pedigree errors and cryptic relatedness in the GAW18 sample using genome-wide SNP data

**DOI:** 10.1186/1753-6561-8-S1-S23

**Published:** 2014-06-17

**Authors:** Lei Sun, Apostolos Dimitromanolakis

**Affiliations:** 1 Division of Biostatistics, Dalla Lana School of Public Health, University of Toronto, Canada; 2 Department of Statistical Sciences, University of Toronto, Canada; 3 Department of Clinical Biochemistry, University Health Network, Canada

## Abstract

Pedigree errors and cryptic relatedness often appear in families or population samples collected for genetic studies. If not identified, these issues can lead to either increased false negatives or false positives in both linkage and association analyses. To identify pedigree errors and cryptic relatedness among individuals from the 20 San Antonio Family Studies (SAFS) families and cryptic relatedness among the 157 putatively unrelated individuals, we apply PREST-plus to the genome-wide single-nucleotide polymorphism (SNP) data and analyze estimated identity-by-descent (IBD) distributions for all pairs of genotyped individuals. Based on the given pedigrees alone, PREST-plus identifies the following putative pairs: 1091 full-sib, 162 half-sib, 360 grandparent-grandchild, 2269 avuncular, 2717 first cousin, 402 half-avuncular, 559 half-first cousin, 2 half-sib+first cousin, 957 parent-offspring and 440,546 unrelated. Using the genotype data, PREST-plus detects 7 mis-specified relative pairs, with their IBD estimates clearly deviating from the null expectations, and it identifies 4 cryptic related pairs involving 7 individuals from 6 families.

## Background

Mis-specified pedigree relationship and cryptic relatedness often occur in family and population data. The potential causes of such errors are numerous, including undocumented nonpaternity, nonmaternity, adoption, mating between relatives, sample duplication or swap. It is well known that such errors, if undetected, can affect the accuracy or power of both linkage and association studies, as well as have adverse effects on other aspects of the analyses such as population stratification [1-6].

Genome-wide marker data can provide accurate information on the genetic relatedness among individuals. For linkage scans, a number of statistical methods have been proposed and implemented, including RELCHECK [1], RELATIVE [7], PEDCHECK [8], SIBERROR [9], PREST [10,11], GRR [12], and ECLIPSE [13], among others. More recently, PLINK [14] and PREST-plus [5,6,15] have been developed for analysing the high-throughput SNP data collected from genome-wide association studies (GWAS) or next-generation sequencing (NGS) experiments.

There are 3 main differences between PREST-plus and PLINK. First, PREST-plus uses the maximum likelihood-based IBD estimation method, which has more statistical power, whereas PLINK relies on the method-of-moments approach, which is computationally more efficient. Second, PREST-plus identifies both pedigree errors and cryptic relatedness in linkage or association studies with family data, population sample, or both, whereas PLINK is primarily suitable for detecting cryptic relatedness in GWAS with population sample. Third, PREST-plus provides a formal hypothesis testing framework, which can be useful when a potential error has been being identified, whereas PLINK provides point IBD estimation only [5,6]. The Genetic Analysis Workshop 18 (GAW18) data, similar to many emerging large-scale GWAS and NGS studies, include multigeneration large pedigrees. The genetic relationships between individuals in such data are not limited to simple types such as parent-offspring or siblings. Consequently, we focus here on the methodology implemented in PREST-plus [5,6,15] and discuss PLINK [14] when relevant.

## Methods

### Relationship estimation and testing

The relatedness between a pair of individuals can be summarized by the IBD probability distribution, $p=\left(p_{0},\;p_{1},\;p_{2}\right)$. It describes the probability of a randomly sampled marker to have 0, 1, or 2 common ancestry alleles between 2 individuals. Using the available genotype data, PREST-plus estimates the most likely IBD distribution by obtaining $\hat{p}=\left({\hat{p}}_{0},\;{\hat{p}}_{1},\;{\hat{p}}_{2}\right)$ that maximizes the quantity

$$L\left(p\right)=\overset{M}{\underset{m=1}{\sum }}\text{log}\left(P\left(G_{m};p\right)\right)=\overset{M}{\underset{m=1}{\sum }}\text{log}\left(\sum_{i=0,1,2}P\left(G_{m}|D_{m}=i\right)p_{i}\right),$$

where $G_{m}$ is the genotype at marker *m *and $D_{m}=i$ denotes the number of alleles shared IBD by the pair at that marker. The maximization is efficiently achieved by an application of the expectation-maximization (EM) algorithm [10,16,17]. In contrast, PLINK [14] estimates the IBD distribution using the method-of-moments approach, which is less powerful than the likelihood-based method.

Estimation of IBD distribution can often provide sufficient information to identify pedigree errors and cryptic relatedness [5,6]. However, it is useful to provide statistical evidence beyond point estimation. To this end, we apply the maximum likelihood ratio test (MLRT) to formally evaluate whether the observed genotypes *G *are compatible with the null putative relationship type, *R_0 _*[10]. Briefly, $MLRT=\text{log}\left(\;\hat{L}\left(A\right)\;\right)-\text{log}\left(\;L\left(R_{0}\right)\;\right),$ where $L\left(R_{0}\right)$ is the likelihood calculated for the hypothesized *R_0_*, and $\hat{L}\left(A\right)\;)=\text{max}\left\{L\left(R_{1}\right)\right\},\;R_{1}\in A,$ is the maximum likelihood calculated over a set of alternatives as given in Table 1. Statistical significance of *MLRT *is then assessed using simulation, because 2*MLRT does not follow the usual *Chisq *distribution [10]. Efficient implementation of the test to high-throughput genotype data requires pruning of the SNPs so that they are not in linkage disequilibrium (LD) [5,6].

### Data analyses

The GWAS data of GAW18 consist of 959 individuals genotyped at 472,049 SNPs. Among the 959 individuals, 4 are removed for low genotyping rate (*plink -MIND >0.8*), and 141 individuals are in the "UNREL.txt" file that contains the maximum set of putatively unrelated individuals. Among the 472,049 SNPs, ~50,000 remain after minor allele frequency (MAF) and LD pruning (*plink -indep-pairwise 200 50 0.2 -maf 0.05*). We then conduct 3 sets of analyses, with analyses 1 and 2 focusing on relationship estimation and analysis 3 performing hypothesis testing.

Analysis 1 detects pedigree errors and cryptic relatedness in the 955 genotyped individuals from the 20 SAFS families using PREST-plus (*prest -geno datafamily.ped -map datafamily.map -wped -aped*). It estimates the IBD distribution for any pair of individuals within a pedigree *(-wped*), as well as for any pair of individuals across pedigrees *(-aped*).

Analysis 2 detects cryptic relatedness in the 141 putatively unrelated individuals, using both PREST-plus (*prest -geno dataunrel.ped -map dataunrel.map*) and PLINK (*plink -file plink -genome*).

Analysis 3 performs formal hypothesis testing on the problematic pairs identified in analyses 1 and 2. For computational efficiency, we first randomly select ~2000 SNPs from the set of ~50,000 SNPs. We then obtain the base pair (bp) physical map of the SNPs using build 36 coordinates and their corresponding centimorgan (cM) genetic map using the Rutgers combined linkage-physical map and linear interpolation. Finally, we perform the MLRT test as implemented in PREST-plus (*prest -file data2k.ped -map data2k.map -pair fID1 indID1 fID2 indID2 -mlrt -c*).

## Results

### Analysis 1: Relationship estimation within and across the 20 SAFS families

**Figure 1:**
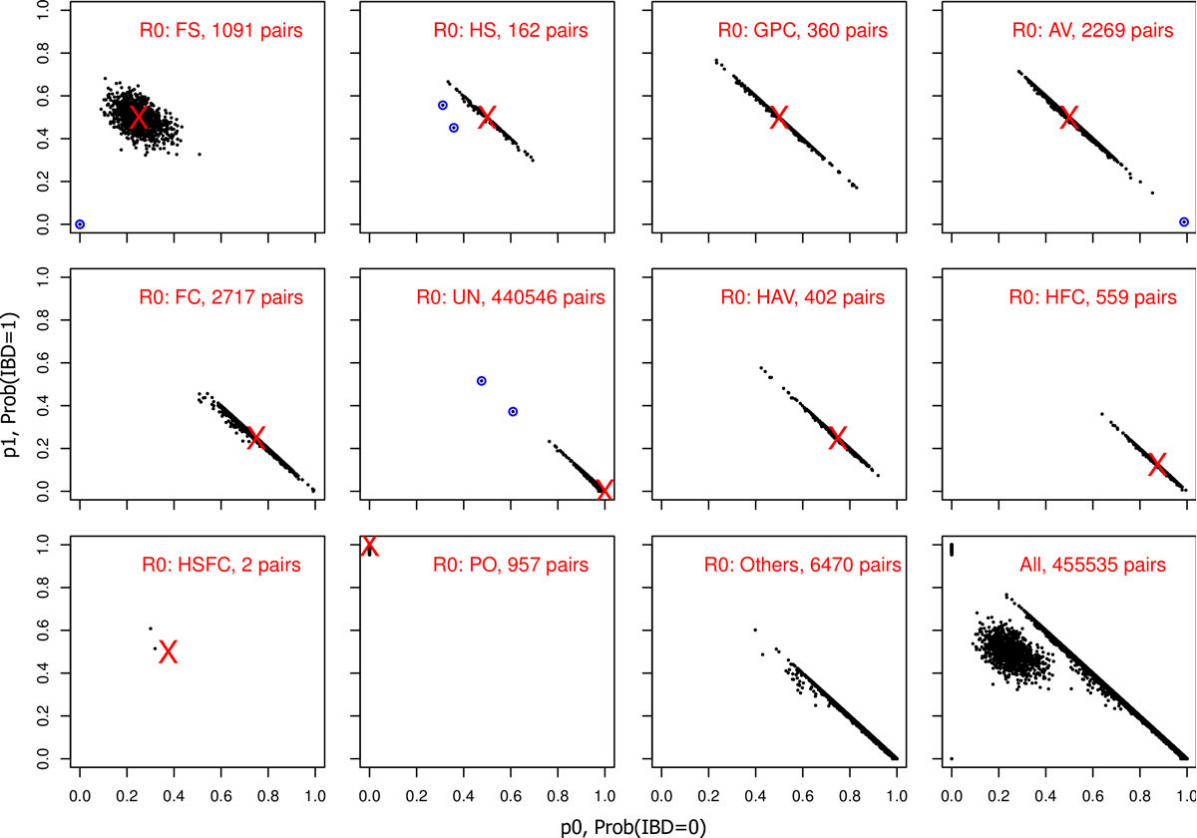
**Results of analysis 1: relationship IBD estimation within and between the 20 SAFS families using PREST-plus**. The figures are stratified by the null putative relationship, *R_0_*, as defined by the given pedigrees. The red cross marks the expected IBD distribution for *R_0 _*as provided in Table 1. Each black dot shows the estimated *p_1 _*vs. *p_0 _*based on the obseved genotype data for each of the 455,535 genotyped pairs analyzed, inlucidng 1091 full-sib, 162 half-sib, 360 grandparent-grandchild, 2269 avuncular, 2717 first-cousin, 440,546 unrelated (from both within and across families), 402 half-avuncular, 559 half-first cousin, 2 half-sib+first cousin, 957 parent-offspring, and 6470 other types of pairs. Blue circles mark the obvious outliers as detailed in Table 2.

PREST-plus identifies 455,535 pairs of genotyped individuals, with the total number of genotyped SNPs (*commark*) ranging from 31,120 to 49,020. Most of the 455,535 pairs have the putative relationship types considered by PREST-plus (see Table 1). (See Figure 1 of reference [5] for graphical illustrations of these relationship types.)

**Table 1:** IBD distribution $p=\left(p_{0},\;p_{1},\;p_{2}\right)$ and kinship coefficient $\phi =p_{1}/4+p_{2}/2$ for the relationship types (reltype) considered by PREST-plus

reltype coding in PREST-plus	Relationship type (abbreviation)	Distribution of IBD sharing			Kinship coefficient, φ
			
		*p_0_*	*p_1_*	*p_2_*	
11	MZ-twin (MZ)	0.000	0.000	1.000	0.50000
10	parent-offspring (PO)	0.000	1.000	0.000	0.25000
1	full-sib (FS)	0.250	0.500	0.250	0.25000
9	half-sib+first cousin (HSFC)	0.375	0.500	0.125	0.18750
2	half-sib (HS)	0.500	0.500	0.000	0.12500
3	grandparent-grandchild (GPC)	0.500	0.500	0.000	0.12500
4	avuncular (AV)	0.500	0.500	0.000	0.12500
5	first cousin (FC)	0.750	0.250	0.000	0.06250
7	half-avuncular (HAV)	0.750	0.250	0.000	0.06250
8	half-first cousin (HFC)	0.875	0.125	0.000	0.03125
6	unrelated (UN)	1.000	0.000	0.000	0.00000
99	other types (Others)	NA	NA	NA	NA

Figure 1 shows the estimated IBD distributions, ${\hat{p}}_{1}$ vs ${\hat{p}}_{0}$, stratified by the putative relationship, *R_0_*, with the red cross marking the the IBD distribution expected for *R_0_*. We observe a substantial number of pairs with their IBD estimates deviating from the null expectations. Table 2 provides detailed information for 7 clear outliers, indicating mis-specified relationships. For example, 2 putative half-sib pairs have close to (0.25, 0.5, 0.25) of full-sib, while 1 putative avuncular pair has close to (1, 0, 0) of unrelated. Analysis 3 below is to determine the statistical significance of the apparent deviation in the IBD estimates.

**Table 2:** Relationship estimation results for clear outliers in Figure 1 identified by analysis 1.

						Estimated		
						
FID1^a^	IID1^b^	FID2^a^	IID2^b^	reltype^c^	commark^d^	*p_0_*	*p_1_*	*p_2_*
3	T2DG0300174	3	T2DG0300175	1	49009	0.0000	0.0000	1.0000
4	T2DG0400281	4	T2DG0400282	1	48996	0.0000	0.0000	1.0000
4	T2DG0400265	4	T2DG0400266	2	48994	0.358	0.4511	0.1909
21	T2DG2100946	21	T2DG2100947	2	48957	0.3112	0.5566	0.1322
21	T2DG2100952	21	T2DG2100966	4	48949	0.9876	0.0109	0.0015
4	T2DG0400207	4	T2DG0400260	6	48955	0.4759	0.5157	0.0084
4	T2DG0400207	4	T2DG0400247	6	47503	0.6094	0.3723	0.0182

These 7 pairs of individuals have their estimated IBD distributions clearly deviating from the null expected values as specified in Table 1.

a Family ID.

b Individual ID.

c Relationship type as in Table 1.

d The number of common markers genotyped for both individuals.

### Analysis 2: Relationship estimation among individuals in the "UNREL.txt" file

**Figure 2:**
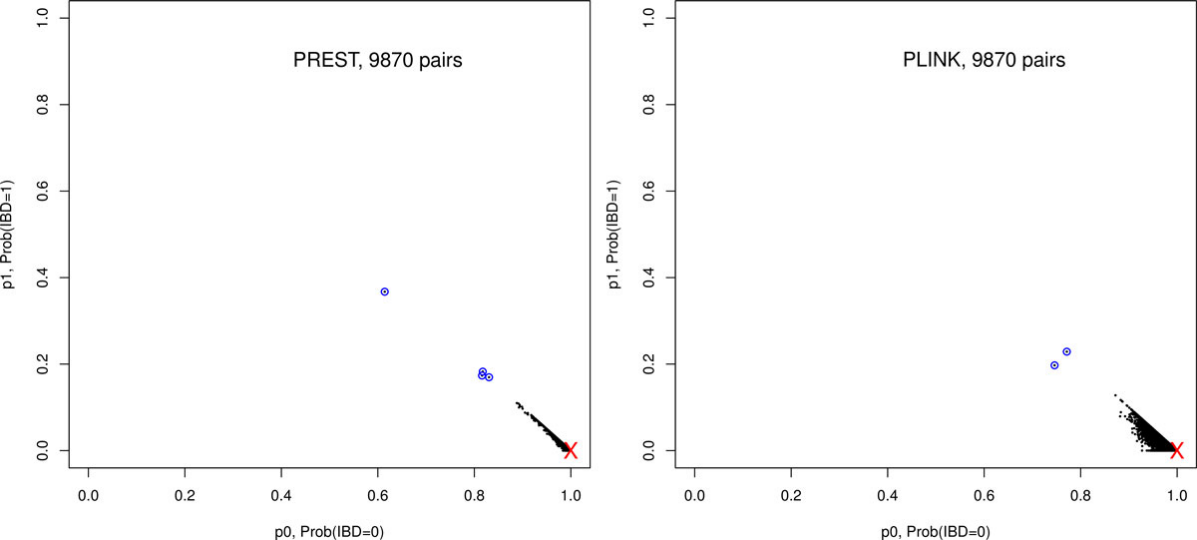
**Results of analysis 2: relationship IBD estimation among the 141 genotyped putatively unrelated individuals in the "UNREL**.txt" file. The red cross marks the the IBD distribution expected for unrelated. Each black dot shows the estimated *p_1 _*vs. *p_0 _*based on the obseved genotype data for each of the 9870 putatively unrelated pairs analyzed by PREST-plus (left) and PLINK(right). Blue circles mark the obvious outliers as detailed in Table 3.

In this analysis, there are 141*140/2 = 9780 putatively unrelated pairs and the *commark *ranges from 32,280 to 49,010. Figure 2 displays ${\hat{p}}_{1}$ vs ${\hat{p}}_{0}$ for these pairs based on PREST-plus (left) and PLINK (right). Results clearly demonstrate the statistical efficiency of PREST-plus with overall less variation in the IBD estimates as compared to PLINK: 9338 pairs with PREST ${\hat{p}}_{0}\geq 0.98$ vs. 6538 pairs with PLINK ${\hat{p}}_{0}\geq 0.98$. For the 4 potential cryptic relateded pairs, the PREST-plus IBD estimates provide a better identification of the outliers (Table 3).

**Table 3:** Relationship estimation results for clear outliers in Figure 2 identified by analysis 2

						PREST-plus estimated			PLINK estimated		
							
FID1	IID1	FID2	IID2	reltype	commark	*p_0_*	*p_1_*	*p_2_*	*p_0_*	*p_1_*	*p_2_*
9	T2DG0901244	10	T2DG1000566	6	48912	0.8159	0.1735	0.0105	1.0000	0.0000	0.0000
8	T2DG0800497	9	T2DG0901244	6	48957	0.8304	0.1696	0.0000	1.0000	0.0000	0.0000
21	T2DG2100951	25	T2DG2501033	6	48940	0.8174	0.1826	0.0000	0.7713	0.2287	0.0000
4	T2DG0400207	4	T2DG0400247	6	47503	0.6142	0.3673	0.0185	0.7460	0.1972	0.0568

These four pairs of individuals have their estimated IBD distributions clearly deviating from the values expected for unrelated pairs.

### Analysis 3: Relationship hypothesis testing of problematic pairs

As a proof of principle, we focus on the 7 clear outliers identified in analysis 1 and the 4 pairs identified in analysis 2 (Table 4). Among the putatively unrelated pairs, the possible alternative relationship types range from half-first cousin to avuncular.

**Table 4:** Relationship testing results for clear outliers in Figure 1 identified by analysis 1, and in Figure 2 identified by analysis 2

FID1	IID1	FID2	IID2	null reltype		*p *value	plausible reltype		*p *value
The 7 outliers identified by analysis 1									
3	T2DG0300174	3	T2DG0300175	1	full-sib	0	11	MZ-twins	N/A
4	T2DG0400281	4	T2DG0400282	1	full-sib	0	11	MZ-twins	N/A
4	T2DG0400265	4	T2DG0400266	2	half-sib	0	9	half-sib+first cousin	0.254
21	T2DG2100946	21	T2DG2100947	2	half-sib	0	9	half-sib+first cousin	0.432
21	T2DG2100952	21	T2DG2100966	4	avunuclar	0	6	unrelated	0.891
4	T2DG0400207	4	T2DG0400260	6	unrelated	0	2	half-sib	0.328
4	T2DG0400207	4	T2DG0400247	6	unrelated	0	5	first cousin	0.752
The 4 outliers identified by analysis 2									
9	T2DG0901244	10	T2DG1000566	6	unrelated	0	8	half-first cousin	0.112
8	T2DG0800497	9	T2DG0901244	6	unrelated	0.007	8	half-first cousin	0.673
21	T2DG2100951	25	T2DG2501033	6	unrelated	0	5	first cousin	0.633
4	T2DG0400207	4	T2DG0400247	6	unrelated	0	5	first cousin	0.712

Empirical *p*-values are based on 25,000 simulated replicates, with genotype data simulated under a specified relationship type. The simulating relationship type can be the null relationship defined by the given pedigrees (i.e. the null reltype) or another relationship type (i.e. the plausible reltype) as listed in Table 1. The possible plausible relationship types are not unique and the table provides the one with the highest *p*-values. Small *p*-value for testing the null reltype suggests that the observed genotype data are not compatible with the null relationship defined by the given pedigree, whereas large *p*-value for testing the plausible reltype suggests that the observed genotype data are compatible with the proposed alternative.

## Discussion

The GAW18 "pedigree information was [previously] verified by estimated kinship coefficients, principal component analysis (PCA), and number of mendelian errors between parent and offspring samples." However, no other details are provided and our analyses show that pedigree errors and cryptic relatedness exist in the data.

The results presented here are based on the ~50,000 GWAS SNPs that have MAF greater than 5% and pair-wise LD less than 0.2. Our experience with PREST-plus shows that there is little improvement in estimation accuracy, once more than ~50,000 SNPs were used (typically with MAF >5%). Additional analyses with denser sets of SNPs confirmed this (results not shown). However, substantially more SNPs are needed for PLINK to achieve similar estimation efficiency. Although PREST-plus is more powerful than PLINK, there is a trade-off between statistical power and computational efficiency [5,6]. For large data sets involving analyzing millions of pairs, PLINK could be used as a screening tool for further analysis with PREST-plus.

Both PREST-plus and PLINK are sensitive to mis-specified allele frequencies, therefore sensitive to population stratification and population admixture, in contrast to some recent work [eg, [18]]. However, results from other GAW18 study groups suggest that population admixture is not a major concern here. Nevertheless, robust relationship estimation and testing methods warrant further research.

The methods considered here focus on global estimation of IBD distribution, which is powerful and efficient to distinguish distinct relationships, for example, full-sibs versus unrelated. However, such global methods are not adequate to distinguish similar relationship types, for example, second-cousin versus unrelated. To this end, the recent local estimation methods [eg, [19]] provide useful research direction.

## Conclusions

Pedigree errors and cryptic relatedness often occur in sample despite the best practice in data collection. Genome-wide marker data, collected for linkage or association studies, can provide accurate genealogy information between individuals. Using the GWAS SNP data, PREST-plus analyses the GAW18 sample that had been previously "cleaned," and it identifies 7 clearly mis-specified relative pairs in the 20 SAFS families and 4 cryptic-related pairs in the set of putatively unrelated individuals.

## Competing interests

The authors declare that they have no competing interests.

## Authors' contributions

Study design: LS. Analysis: AD and LS. Manuscript drafting: LS and AD. All authors read and approved the final manuscript.
